# Exponential Megapriming PCR (EMP) Cloning—Seamless DNA Insertion into Any Target Plasmid without Sequence Constraints

**DOI:** 10.1371/journal.pone.0053360

**Published:** 2012-12-31

**Authors:** Alexander Ulrich, Kasper R. Andersen, Thomas U. Schwartz

**Affiliations:** 1 Department of Biology, Massachusetts Institute of Technology, Cambridge, Massachusetts, United States of America; 2 Institut für Chemie und Biochemie, Freie Universität Berlin, Berlin, Germany; Center for Genomic Regulation, Spain

## Abstract

We present a fast, reliable and inexpensive restriction-free cloning method for seamless DNA insertion into any plasmid without sequence limitation. Exponential megapriming PCR (EMP) cloning requires two consecutive PCR steps and can be carried out in one day. We show that EMP cloning has a higher efficiency than restriction-free (RF) cloning, especially for long inserts above 2.5 kb. EMP further enables simultaneous cloning of multiple inserts.

## Introduction

One of the most common tasks in molecular cloning is the insertion of a defined DNA fragment into a target plasmid. Traditionally this is achieved by restriction enzyme mediated sticky end [1]–[4] or blunt end [5]–[6] ligation, greatly facilitated by the advent of PCR [7]–[9]. Nonetheless the efficiency of blunt end ligation is low and sticky end cloning is limited by the availability of suitable restriction sites. To overcome these disadvantages restriction-free cloning techniques have been developed, albeit all with their own limitations. TA-cloning [10] needs special vector treatment and does not discriminate against reverse insertion of the insert. The high-throughput methods Gateway [11]–[12] and Creator cloning [13] use site-specific recombination, thus depend on specific sequence elements and, in addition, require specific vectors and expensive enzymes. In contrast, homologous recombination techniques rely on fusion of complementary sequences and thus do not need specific sequence elements. *In vivo* homologous recombination (reviewed in [14]) can be achieved by three mechanisms, all limited in one way or another. RecA-dependent recombination [15] requires *recA*+ strains and long overhangs, RecA-independent recombination [16] is of low efficiency and Red/ET dependent recombination [17] depends on strains overexpressing RedE/RedT/Redγ. Homologous recombination *in vitro* does not require special bacteria strains. Instead recombination is facilitated by *in vitro* generation of single strand overhangs. For this purpose ligation-independent cloning (LIC) [18] uses the 3′–5′ exonuclease activity of T4 DNA polymerase. Overhangs are typically generated from ∼12 nt terminal sequences lacking one of the four nucleotides. The sequence restriction is necessary to avoid uncontrolled DNA digestion. In the case of sequence and ligation-independent cloning (SLIC) [19] overhangs are determined either by stopping the exonuclease reaction after a certain time, or by PCR. SLIC generated overhangs have no sequence restrictions other than being complementary to the target plasmid. SLIC requires linearizing the target plasmid by enzymatic cleavage or PCR, and the addition of RecA, for highest efficiency. Another recent *in* *vitro* recombination technique was presented by Gibson and coworkers [20]. Here double stranded DNA fragments of up to several hundred kilobases with overlapping sequences of 40 bp are assembled in a single reaction using 5′ exonuclease, DNA polymerase and DNA ligase. This method can be used for the assembly of genes and entire genomes. A common weakness of all recombination-based cloning techniques is that cloning success cannot be monitored before obtaining colonies, since the intermediary steps are not quantifiable. A megaprimer-based method, restriction-free (RF) cloning [21]–[23], is also sequence-independent and restriction-free, like SLIC, but in addition does not require enzymatic strand treatment and intermediary steps can be monitored and controlled. Traditionally, the megaprimer PCR method was used to introduce mutations, insertions and deletions into a linear DNA sequence [24]–[25] or to fuse DNA fragments [26]. In RF cloning the insert is amplified with primers containing overhangs matching a sequence of choice in the target plasmid. In a second PCR reaction the PCR product of the first reaction is used as a megaprimer for linear amplification of the target plasmid. The resulting product can be observed via agarose gel electrophoresis and, in case of success, be transformed. This method works reasonably well for inserts up to 5 kb in length, although in practice efficiency is reduced for inserts >2–3 kb. A major disadvantage of RF cloning is low product yield due to linear PCR amplification, which becomes prohibitive for larger inserts.

**Figure 1:**
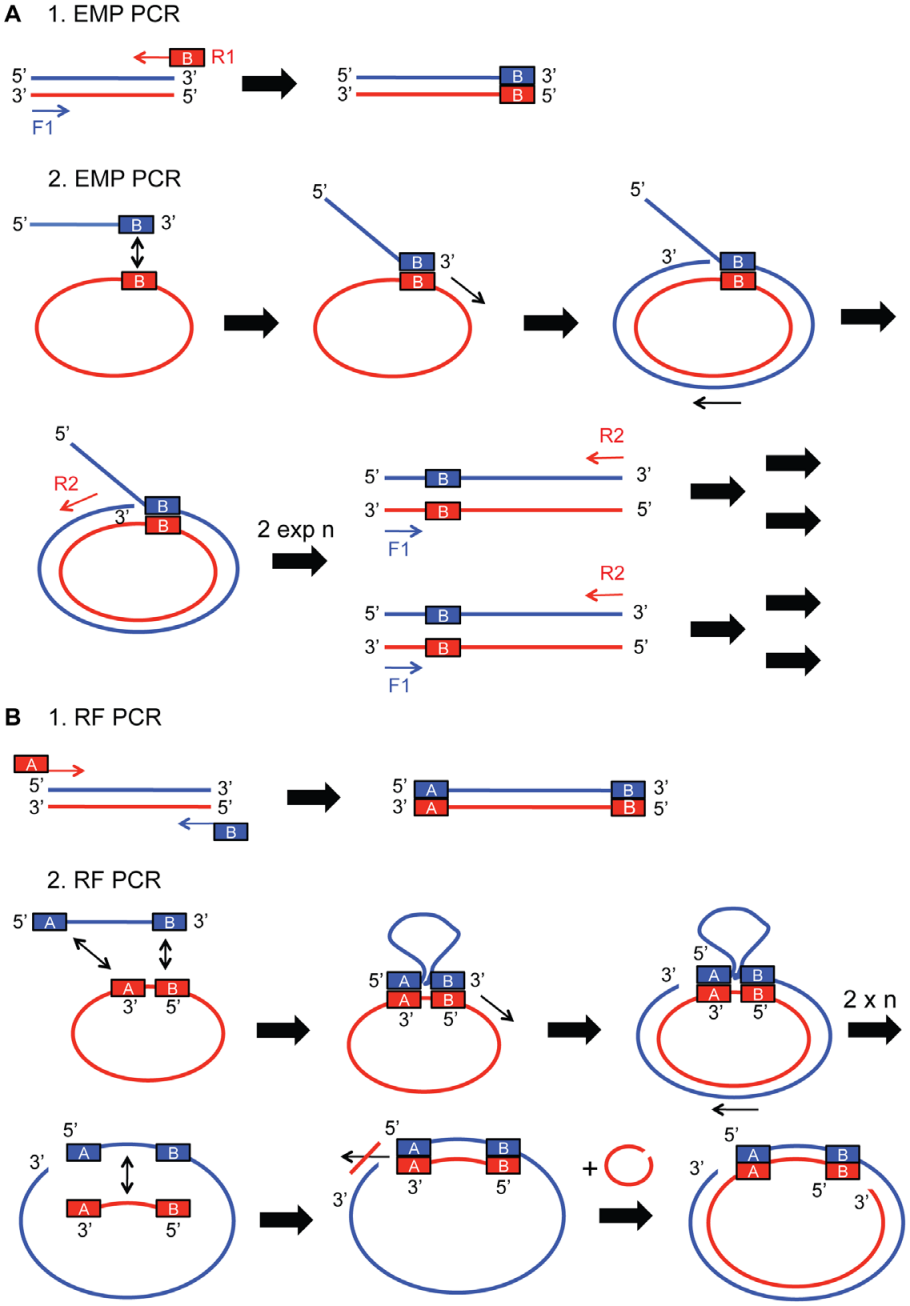
Mechanisms of EMP and RF cloning. Mechanistic details of EMP and RF cloning are compared. (A) EMP cloning involves two PCR reactions. In the 1^st^ EMP PCR a forward primer F1 and a reverse primer R1 with overhang exponentially amplify the insert of interest. In the 2^nd^ EMP PCR reaction the purified product is used as a megaprimer to exponentially amplify the target plasmid together with a forward primer F1 and reverse primer R2. (B) RF cloning also includes two PCR reactions. In the 1^st^ RF PCR two overhang containing primers F1 and R1 exponentially amplify the insert of interest. In the 2^nd^ RF PCR the purified product is used as a megaprimer to linearly amplify the target plasmid. Experimental procedures are described in detail in **Materials and Methods** and **Tables S1** and **S2**.

To overcome the limitations of current cloning methods, we developed exponential megapriming PCR (EMP) cloning. EMP cloning shares the advantages of RF cloning, but lifts the size limits for the inserts. EMP requires two consecutive PCR steps, which are both designed to amplify the template exponentially rather than linearly. In the first step, the insert is amplified, and in the second step the insert is integrated into the plasmid (**Fig. 1A**). Both steps can also be combined into a single PCR reaction. Importantly, simultaneous insertion of several DNA fragments is possible with EMP cloning.

## Materials and Methods

### Exponential Megapriming PCR (EMP) Cloning

#### Overview of EMP cloning

The first step of EMP cloning is the exponential amplification of the insert of choice and the addition of a 3′ overhang by PCR. The product of this 1^st^ EMP PCR is used in a second PCR reaction as a megaprimer to exponentially amplify the target plasmid together with a short reverse primer (**Fig. 1A**). The product of this 2^nd^ EMP PCR is then *in vitro* phosphorylated, ligated and transformed into competent *E. coli* cells. Single colonies are screened for proper plasmid generation by standard methods, i.e. analytical restriction digest, colony-PCR, or by sequencing.

#### Synthesis of the EMP megaprimer

For the synthesis of the megaprimer two primers are used in an exponential PCR reaction (1^st^ EMP PCR). The forward primer (F1) typically has 20–25 nt and matches the 5′ region of the insert. The reverse primer (R1) is 40–50 nt in size and contains a 20–25 nt sequence with reverse complementarity to the region on the target plasmid immediately downstream of the insertion site, followed by the reverse complement of the 3′ end of the insert. The 1^st^ EMP PCR reactions contains 1× HF Phusion buffer (NEB), 200 µM of each dNTP, 0.5 µM primer F1, 0.5 µM primer R1, 25 ng template DNA, and 0.02 U/µL Phusion DNA Polymerase (NEB) in a volume of 50 µl. In rare cases GC Phusion buffer and/or the addition of up to 3% (v/v) dimethylsulfoxide (DMSO) can increase the reaction efficiency. The PCR conditions are: initial denaturation step (30 s, 98°C), followed by 25 cycles of denaturation (10 s, 98°C), annealing (30 s, Tm (F1/R1) +3°C) and extension (15 s/1 kb, 72°C). A final extension cycle (5 min, 72°C) completes the PCR reaction. For the annealing temperature the lower of the calculated melting temperature of either F1 or the insert binding part of R1 is used. Tm values were calculated using OligoAnalyzer 3.1 (IDT). Product of the 1^st^ EMP PCR is analyzed by agarose gel electrophoresis and purified with a PCR purification kit (E.Z.N.A. Cylce Pure Kit, Omega bio-tek). See also **Table S1**.

#### Insertion of the EMP megaprimer

The megaprimer is used together with a 20–25 nt primer R2, reverse complementary to the region 5′ of the insertion site on the target plasmid, to exponentially amplify the target plasmid. The amount of megaprimer is not critical and can be fairly low (25–400 ng), since the re-use of primer F1 again in the 2^nd^ EMP PCR ensures high product yield. In the first cycles the megaprimer and R2 generate a starting population of insert fused with target plasmid. After the megaprimer is depleted, primers F1 and R2 continue to exponentially amplify the linear product. The 2^nd^ EMP PCR contains 1× HF Phusion buffer, 200 µM of each dNTP, 0.5 µM primer F1, 0.5 µM primer R2, 25 ng–400 ng megaprimer, 25 ng template DNA, and 0.02 U/µL Phusion DNA Polymerase (NEB) in a volume of 50 µl. The amount of megaprimer should be screened. If F1 is added to the reaction 25–50 ng megaprimer are sufficient. In rare cases the addition of F1 leads to secondary PCR products and thus low product amounts. In those cases no F1 and megaprimer amounts between 100–400 ng should be tried, with the highest success rate at 200 ng in our tests. PCR optimization is performed as described for the 1^st^ EMP PCR. The 2^nd^ EMP PCR starts with an initial denaturation step (30 s, 98°C), followed by 25 cycles of denaturation (10 s, 98°C), annealing (30s, Tm (F1/R1/R2) +3°C) and extension (30 s/1 kb, 72°C) with no final extension. For the calculation of the annealing temperature the lower Tm of either R2, F1, or the plasmid binding sequence of the megaprimer is used. The product of the 2^nd^ EMP PCR is analyzed by agarose gel electrophoresis and purified with a PCR purification kit. The product is eluted in 30 µl of 10 mM Tris-HCl (pH 8.0), 1 mM EDTA. See also **Table S1**.

#### In vitro ligation and transformation of EMP product

16.5 µl product of the 2^nd^ EMP PCR is incubated in 1× T4 DNA ligase buffer (NEB) with 5 U T4 PNK for 30 min at 37°C to add a 5′ phosphate. The product is circularized by ligation with 200 U of T4 DNA ligase for 1 h at room temperature. Remaining parental plasmid is digested by adding 10 U DpnI for 30 min at 37°C. 5 µL of the 20 µL reaction are used for the transformation into 50 µL of chemically competent *E. coli* DH5α cells.

### One-step EMP Cloning

EMP cloning allows to couple megaprimer production and insertion into the target plasmid in a single PCR reaction. In a one-step EMP PCR reaction limiting amounts of the overhang containing primer R1 are used to reach primer depletion in the first cycles. The resulting small amount of megaprimer generates a starting population of product template for the exponential amplification through forward primer F1 and reverse primer R2. A typical reaction contains 0.02 µΜ πριμερ Ρ1, 0,5 µΜ πριμερ Φ1 ανδ 0.5 µΜ πριμερ Ρ2 ιν αδδιτιον το 1× HF Phusion buffer, 200 µM of each dNTP, 25 ng template DNA for the insert, 25 ng template DNA for the target plasmid, and 0.02 U/µL Phusion DNA Polymerase (NEB) in a 50 µΛ ρεαχτιον. See also **Table S2**.

### Multi-insert EMP Cloning

EMP cloning allows for insertion of several megaprimers at once. The first step of EMP cloning is performed independently for each megaprimer. In the 2^nd^ PCR step 50 ng of each megaprimer are added to 1× HF Phusion buffer, 200 µM of each dNTP, 0.5 µM primer F1, 0.5 µM primer R2, 25 ng template DNA, and 0.02 U/µL Phusion DNA Polymerase (NEB) in a 50 µΛ ρεαχτιον. Megaprimer 3′ overhangs are designed such that they bind, in nested manner, the 5′ end of another insert. The terminal insert then binds to the plasmid backbone 3′ of the insertion site. In an exponential amplification reaction forward primer F1 anneals to the beginning insert ensuring that all inserts are getting amplified. Reverse primer R2 binds 5′ of the insertion site. *In vitro* ligation and transformation are performed as in regular EMP cloning. See also **Table S3**.

### Restriction-free (RF) Cloning

#### Overview RF cloning

The protocol is modified from the original protocol published in van den Ent and Löwe [22]. In the first step of RF cloning the insert of choice gets amplified and overhangs at 3′ and 5′ end are added. The product of this 1^st^ RF PCR is used in a 2^nd^ PCR as a megaprimer to linearly amplify the target plasmid (**Fig. 1B**). The product of this 2^nd^ RF PCR can either be directly transformed, or first *in vitro* ligated and then transformed in competent *E. coli* cells. Single colonies are picked, grown and control digests are performed.

#### Synthesis of the RF megaprimer

For the synthesis of the megaprimer two 40–50 nt primers are used in an exponential PCR reaction (1^st^ RF PCR). The forward primer (F1) has a 20–25 nt region identical with the sequence 5′ of the insertion site of the target plamsid, followed by 20–25 nt matching the 5′ end of the insert. The reverse primer (R1) is also 40–50 nt long and contains 20–25 nt in reverse complementarity to the sequence 3′ of the insertion site of the target plasmid, followed by the reverse complement of the 3′ end of the insert. The resulting product is the exponentially amplified insert containing 5′ and 3′ overhangs and its complementary strand. Besides the two primers the 1^st^ RF reaction has the same reaction conditions as the 1^st^ EMP PCR. Optimization is performed as for the 1st EMP PCR. For the calculation of the annealing temperature the lower Tm of either the insert binding part of F1 or the insert binding part of R1 is used. The product of the 1^st^ RF PCR is analyzed by agarose gel electrophoresis and purified with a PCR purification kit. See also **Table S4**.

#### Insertion of the RF megaprimer

The megaprimer is used to linearly amplify the target plasmid. The overhangs of the megaprimer bind 5′ and 3′ of the insertion site on the target plasmid. A new product strand does not contain a binding site for the reverse megaprimer and is therefore not a template for the next round of PCR, causing a linear rather than exponential amplification. The 2^nd^ EMP PCR contains 1× HF buffer (NEB), 200 µM of each dNTP, 100 ng–400 ng megaprimer, 25 ng template DNA, and 0.02 U/µL Phusion DNA Polymerase (NEB). The amount of megaprimer has to be screened. PCR optimization is performed as for the 1^st^ PCR reaction. The 2^nd^ RF PCR reaction starts with an initial denaturation step (30 s, 98°C), followed by 35 cycles of denaturation (10 s, 98°C), annealing (30s, Tm (F1/R1) +3°C) and extension (30 s/1 kb, 72°C) with no final extension. The product of the 2^nd^ RF PCR is analyzed by agarose gel electrophoresis and purified with a PCR purification kit. See also **Table S4**.

#### Ligation and transformation of the 2^nd^ RF PCR product

Van den Ent and Löwe [22] suggest to directly add DpnI to the finished 2^nd^ RF PCR reaction and incubate for 2 h to digest parental plasmid, followed by transformation in *E. coli* cells. Since the two complementary product strands can form a circular double stranded plasmid with two single strand nicks *in vivo ligation* can occur in E. *coli* cells, albeit with low efficiency.

We instead performed an *in vitro* ligation by incubating the purified 2^nd^ RF PCR product in T4 ligase buffer (NEB) with 5 U PNK for 30 min at 37°C, followed by 1 h incubation at room temperature with 200 U of T4 ligase and incubation for 30 min at 37°C with 10 U DpnI. The product is then transformed in *E. coli* DH5α cells.

The advantage of direct DpnI incubation and transformation of the 2^nd^ RF PCR reaction, as suggested in [22], is to save time and labor by not performing DNA purification, PNK and T4 ligase incubation. However, the *in vivo* ligation protocol has severe disadvantages. First, the efficiency of DpnI in PCR buffer is reduced compared to T4 ligase buffer or NEB buffer 4, resulting in higher background due to incompletely digested parental plasmid. Second, *in vivo* ligation of doubly nicked plasmids is inefficient.

To better compare EMP cloning to RF cloning by eliminating influences of differential product treatment after 2^nd^ PCR we decided to perform RF cloning with *in vitro* ligation.

### General PCR Optimization

Difficulties during DNA amplification by PCR can arise from the nucleotide sequence of primers and template. Secondary structure motifs, such as intramolecular hairpins, in the primer sequence can inhibit annealing to the template. Formation of primer homo-dimers or hetero-dimers dilute the effective primer concentration and, if primers get extended, also the effective polymerase concentration and nucleotide concentration. These problems can be minimized by a careful primer design [27]–[28]. Tools for designing primers are available online. Other reasons for PCR failure are GC-rich sequences and base pair repeats in the DNA template [29]–[30]. GC-rich sequences can lead to the formation of stable, non-B form secondary structure motifs. The ability of the polymerase to amplify difficult templates can be improved by optimizing the PCR buffer by adding Mg^2+^, DMSO, glycerol or formamide [29]–[30] or using commercial buffers for GC-rich sequences. A general way to trouble-shoot PCR reactions is to vary the primer annealing temperature. Initially an annealing temperature close to the lower melting temperature of the used primers should be tested. If this leads to low product amounts a lower annealing temperature should be tried to allow primer binding. If it results in bands of unwanted products a higher annealing temperature should be tried to avoid unspecific priming [31]–[32]. PCR errors by the polymerase can be reduced to a minimum by using high fidelity polyermases such as Phusion Polymerase or Pfu Turbo II instead of Taq polymerase [33]–[36] and by reducing the number of PCR cycles. PCR error rates can be calculated with web tools like the fidelity calculator by Thermo Fisher (http://www.thermoscientificbio.com/webtools/fidelity/).

## Results

### The Concept and Mechanism of Exponential Megapriming PCR (EMP) Cloning

**Figure 2:**
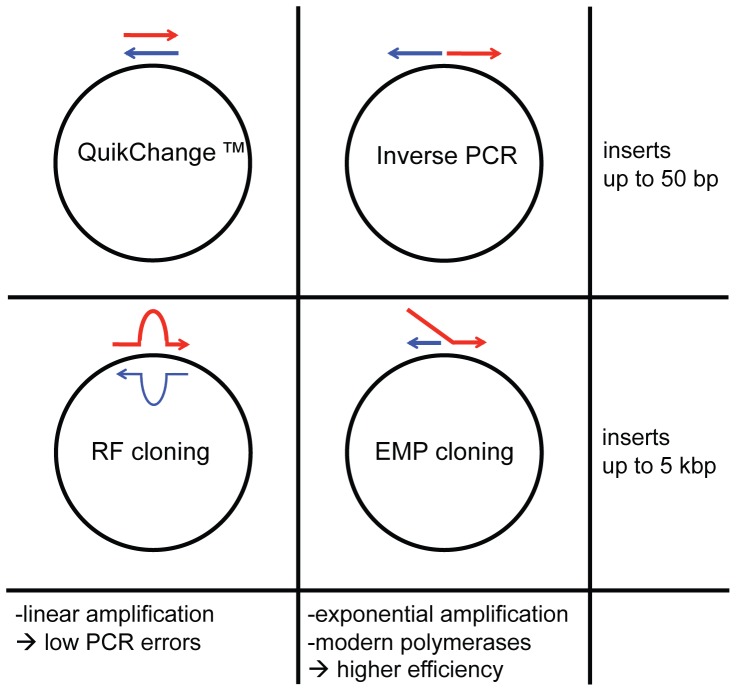
Analogy between PCR-based cloning techniques. QuikChange and inverse PCR allow insertion of up to 50 bp, whereas RF and EMP cloning can accommodate inserts of up to 5 kb. QuikChange and RF cloning use linear amplification to obtain their product. This causes few PCR errors since potential mutations cannot be inherited in the next PCR cycle but also causes low product amounts. Inverse PCR and EMP cloning utilize exponential amplification to obtain high product amounts resulting in intensive, easily observable product bands, and large colony numbers.

The *in vitro* mutagenesis PCR method QuikChange [37]–[38] is the conceptual basis for RF cloning, just that it introduces single mutations or small insertions or deletions <50 bp instead of entire genes. In the QuikChange PCR reaction a complementary primer pair binds to the target site leading to linear product amplification. Although the efficiency of these short manipulations is fairly high, PCR products can often not be observed due to low product amounts. In analogy to how RF cloning relates to QuikChange, EMP cloning relates to inverse PCR (iPCR) [39] (**Fig. 2**). iPCR can be used for the same manipulations QuikChange was developed for, but it uses non-overlapping primers for exponential target amplification, resulting in drastically higher product amounts and ultimately more positive clones. iPCR was originally problematic due to the relatively high error-rate of native, thermostable DNA polymerases, such as Taq. Since the introduction of genetically engineered polymerases with extraordinarily low error-rates, such as Phusion and PfuUltra II, there is no longer a disadvantage of PCR amplifying large pieces of DNA, such as vector backbones [33]–[36]. EMP cloning now uses the mechanism of iPCR to introduce complete genes.

In EMP cloning the insert is amplified with a forward primer (F1) without and a reverse primer with overhang (R1) (**Fig. 1A**). This leads to a product with 3′ overhang complementary to a landing sequence downstream of the desired insertion site in the plasmid. In the second PCR reaction the overhang binds to the target plasmid at the landing site and a second short primer (R2) binds upstream of the insertion site, mimicking the two primers of an iPCR reaction, and resulting in exponential product amplification. After megaprimer depletion the re-used primer F1 and primer R2 continue to exponentially amplify the linear product. This allows relatively low amounts of megaprimer (25 ng) (**Fig. 1A**, **Table S1** and **Materials and Methods**). In RF cloning in comparison, the first PCR reaction uses two overhang containing primers (F1 and R1) to amplify the insert and to create a megaprimer with two overhangs. In the second PCR this megaprimer binds 3′ and 5′ of the insertion site thus product amplification is linear. (**Fig. 1B**, **Table S4** and **Materials and Methods**).

**Figure 3:**
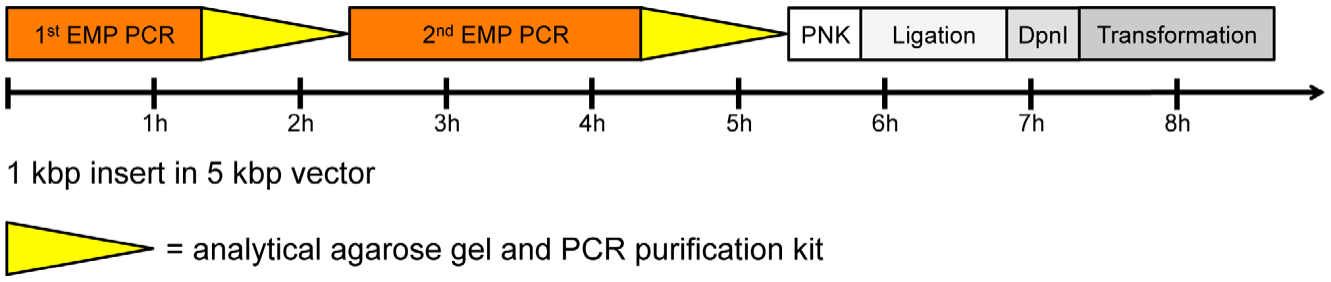
Time scale of a complete EMP cloning experiment. The time scale of a typical EMP cloning experiment with a 1 kb insert and a 5 kb template vector is shown. Boxes indicate separate subroutines of the experiment. Triangles indicate a PCR product analysis and purification step.

EMP cloning follows a simple protocol leading to bacterial colonies in one day (**Fig. 3**). The products of the 1^st^ EMP PCR and 2^nd^ EMP PCR can be analyzed on an agarose gel and purified with a PCR purification kit. The final product needs to be 5′ phosphorylated with T4 Polynucleotide Kinase (PNK), ligated with T4 Ligase and incubated with the restriction enzyme DpnI to digest the parental plasmid. These three enzymatic steps take about 2 h. The product is then transformed into competent *E. coli* cells and colonies are obtained the next day.

### Comparison of EMP Cloning and RF Cloning

**Figure 4:**
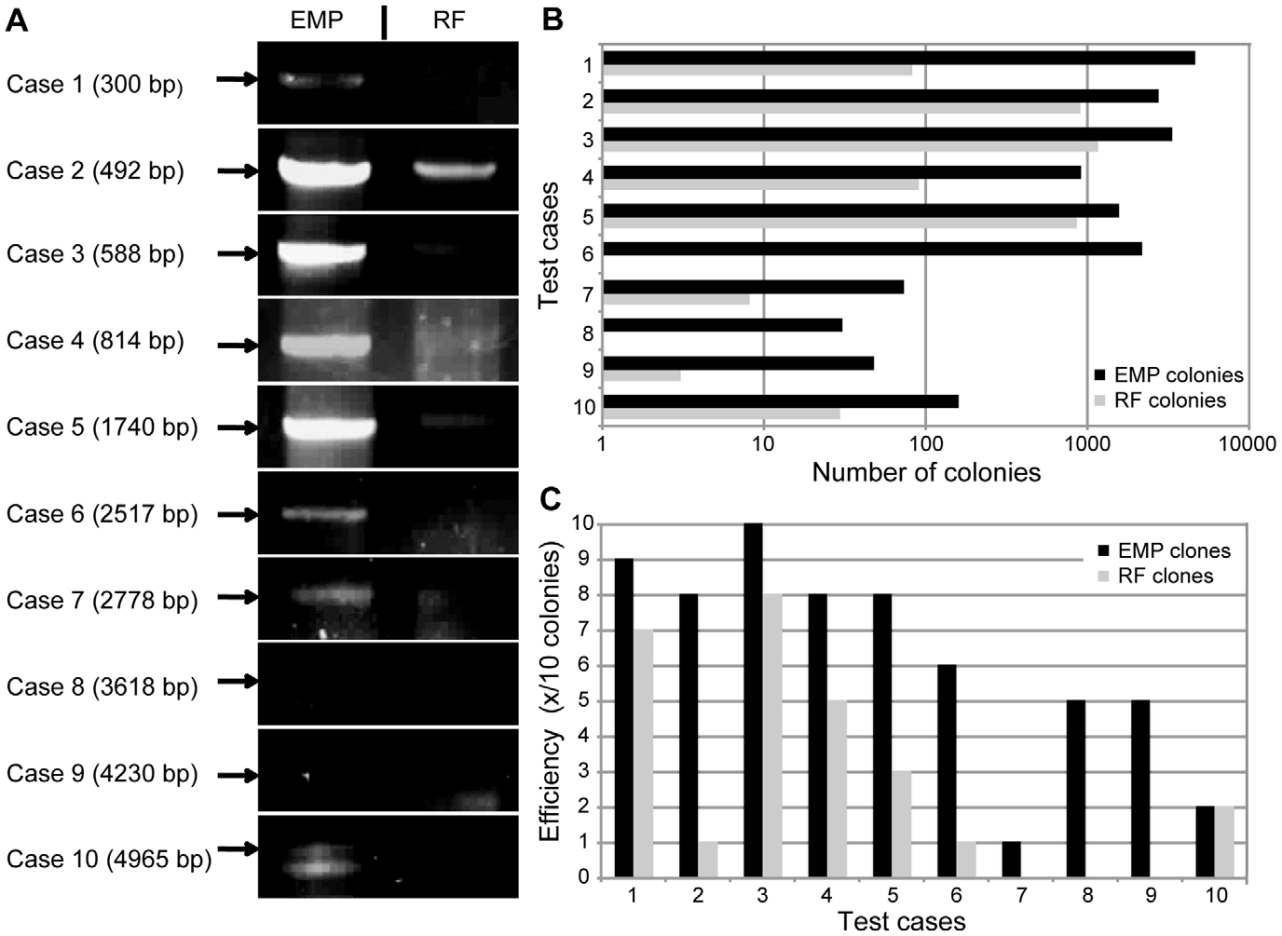
Cloning efficiency of EMP and RF cloning. Cloning efficiency of EMP and RF cloning are compared. (A) Agarose gel band intensities of 2^nd^ EMP and 2^nd^ RF PCR reactions of 10 test cases are compared (insert length in parentheses). Complete agarose gels are shown in **Figure S1**. (B) The number of colonies obtained with EMP and RF cloning are compared (notice that the axis is logarithmic). (C) The cloning efficiency, depicted in number of correct clones of 10 analyzed, is compared for EMP and RF cloning. Agarose gels of restriction digests are shown in **Figure S2**.

To test the performance of EMP cloning we compared it directly to RF cloning. We designed 10 test cases with insert lengths from 0.3 to 5 kb, resulting in plasmids of 3.9 to 13.6 kb (**Table 1**). First we compared the product intensities of the 2^nd^ PCR (**Fig. 4A** and **Fig. S1**). Expectedly, EMP cloning consistently and reproducibly yielded more product than RF cloning, which can then be monitored more easily by gel electrophoresis. Higher product amounts let to more colonies (**Fig. 4B**). EMP yielded ∼1500 colonies whereas RF about 5-fold less (∼300 colonies). For inserts >2.5 kb the colony number dropped in both methods. Whereas EMP cloning still produced ≥ 30 colonies, for RF cloning the number of colonies dropped to an average of 8, and in three cases there were ≤ three colonies. This drastically reduces cloning efficiency in the RF setup.

**Table 1:** Plasmid length, insert length and cloning efficiency of the 10 test cases.

Case	Insert length (bp)	Template plasmidlength (bp)	Product plasmidlength (bp)^a^	EMP: numberof colonies^b^	RF: numberof colonies^b^	EMP: positive clonesout of 10 clones^c^	RF: positive clonesout of 10 clones^c^
1	300	3741	3909	4552	81	9	7
2	492	7615	8104	2704	888	8	1
3	588	7483	7525	3280	1144	10	8
4	814	7414	7598	896	89	8	5
5	1740	7881	9614	1540	844	8	3
6	2517	3741	6126	2136	1	6	1
7	2778	5904	8676	72	8	1	0
8	3618	5904	9516	30	0	5	0
9	4230	5904	10128	47	3	5	0
10	4965	10220	13656	157	29	2	2

a The sum of insert length and template plasmid length does not automatically equal the product length since some inserts replace parts of the template.

b Number of colonies obtained in a single cloning experiment.

c Clones with the correct digestion pattern out of 10 analyzed clones.

To compare the cloning efficiency, we isolated plasmids of 10 colonies per experiment, if available, and did a control restriction digest (**Fig. 4C** and **Fig. S2**). Compared to RF cloning, the efficiency of EMP cloning was better in 9 cases, and equal in one case. On average the EMP cloning efficiency was more than twice as high as RF cloning. Again the advantage of EMP cloning versus RF cloning is especially pronounced for inserts > 2.5 kb. EMP cloning worked in all cases, RF cloning failed in three cases.

### One-step Reaction and Insertion of Multiple Inserts

EMP cloning is suitable for coupling of megaprimer production and insertion in a single reaction and for adding several inserts into a plasmid simultaneously.

**Figure 5:**
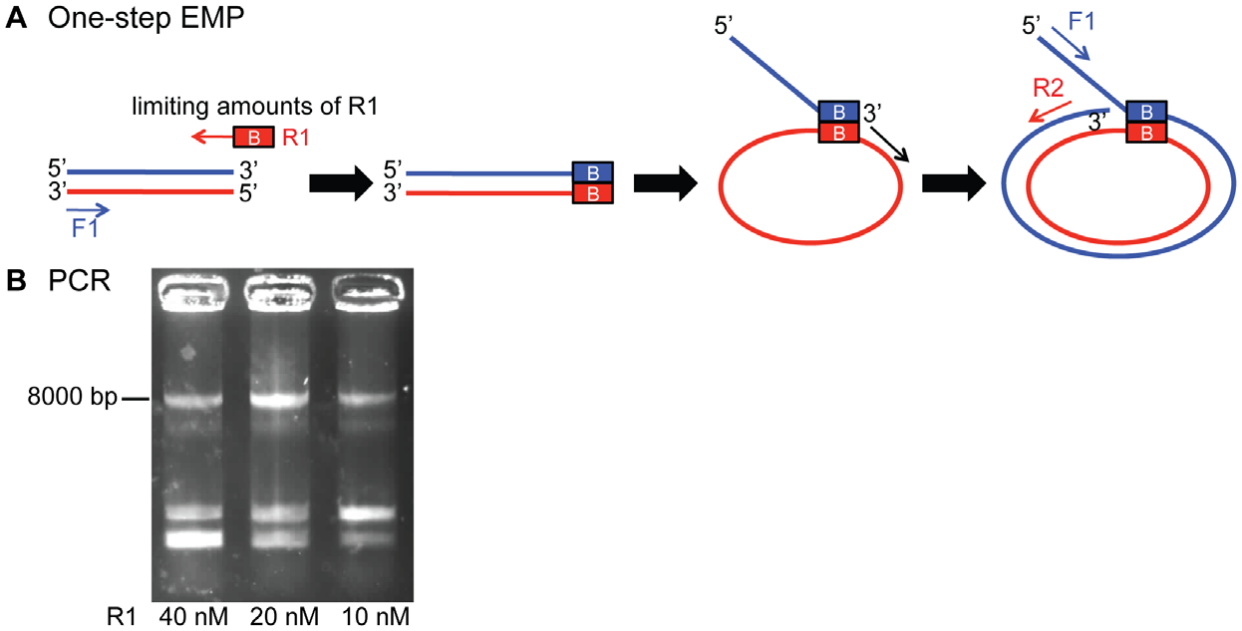
Applications of EMP cloning: One-step EMP. EMP cloning allows for megaprimer production and insertion in a single PCR reaction. (A) In a one-step EMP PCR reaction limiting amounts of the overhang containing primer R1 are used to reach primer depletion in the first cycles. The resulting small amount of megaprimer generates a starting population of product template for the exponential amplification through forward primer F1 and reverse primer R2. (B) Product bands of one-step EMP are compared. 20 nM of primer R1 results in the most intense product band at ∼8 kb.

In order to further improve time efficiency of EMP cloning experiments we developed a one-step protocol for EMP. The experiment can be shortened by combining the 1^st^ and 2^nd^ EMP PCR in one reaction, followed by one purification. One-step EMP cloning uses limited amounts of overhang containing primer (R1). This generates a small population of megaprimers, sufficient to initiate the production of the desired product plasmid. Primer F1 and R2 then exponentially amplify the product plasmid (**Fig. 5A** **, Table S2** and **Materials and Methods**). One-step EMP cloning was tested by inserting a 492 bp fragment into a vector using three different amounts of R1 primer. The PCR reaction worked in all cases (**Fig. 5B**) and correct clones were obtained (**Fig. S3**).

**Figure 6:**
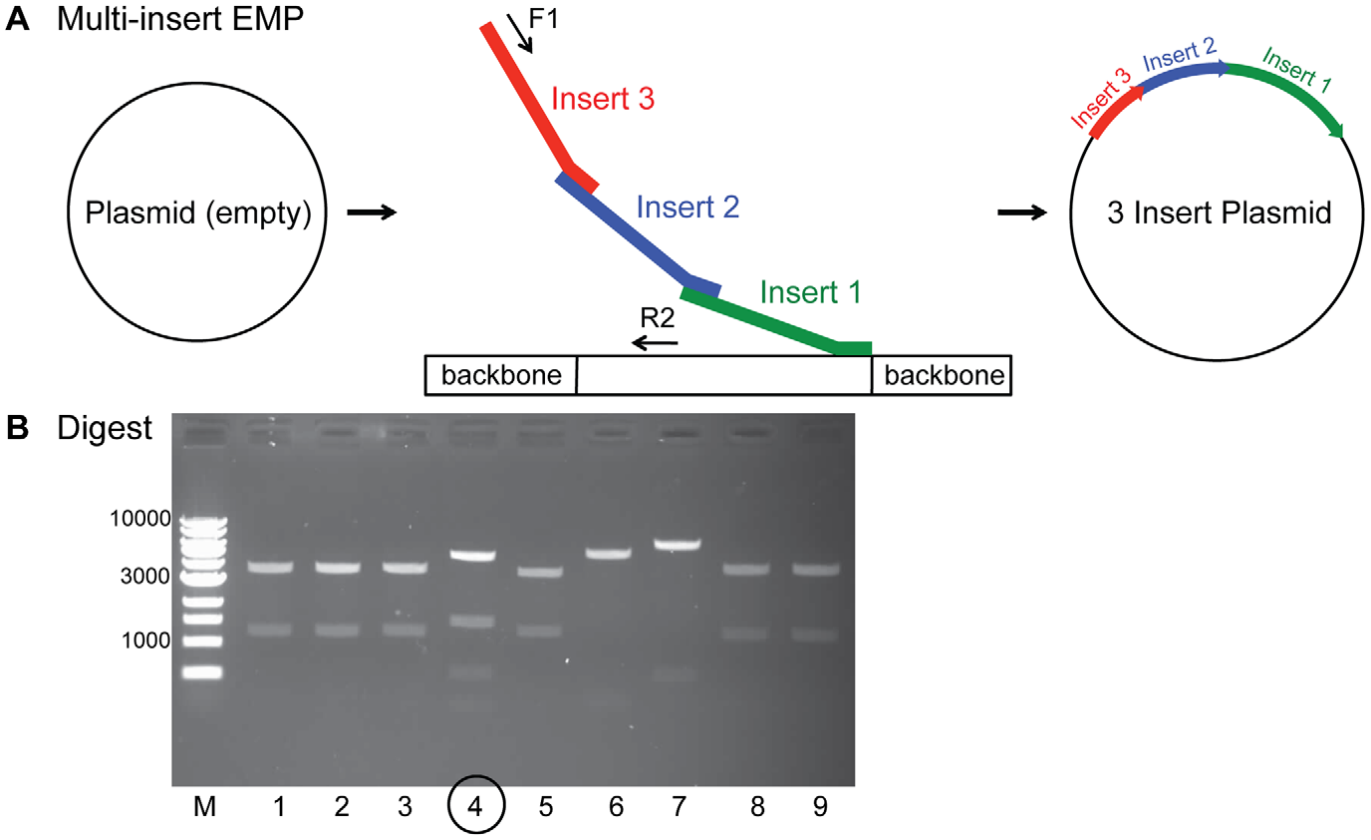
Applications of EMP cloning: Multi-insert EMP. EMP cloning allows for insertion of several megaprimers at once. (A) In a multi-insert EMP megaprimer 3′ overhangs are designed such that they bind in nested manner. The terminal insert (insert 1) then binds to the plasmid backbone 3′ of the insertion site. In an exponential amplification reaction forward primer F1 binds to the beginning insert (insert 3) ensuring that all inserts are getting amplified. Reverse primer R2 binds 5′ of the insertion site. (B) A test digest of nine colonies obtained by multi-insert EMP of three inserts shows one correct clone (number 4).

EMP is an ideal tool for efficient and precise plasmid assembly. To make EMP an even more suitable tool for demanding cloning procedures we developed a multi-insert EMP protocol, enabling simultaneous insertion of several consecutive DNA fragments into a vector (**Fig. 6A**). This is useful for the generation of plasmids for co-expression of proteins. Multi-insert EMP assembles independently produced megaprimers in a single reaction. Megaprimer 3′ overhangs are designed such that they bind in nested manner. The terminal insert then binds to the plasmid backbone downstream of the insertion site. Again Primer F1 and R2 are used to exponentially amplify the product plasmid, where primer F1 binds to the 5′ end of the first insert ensuring that all inserts are getting amplified. The reverse primer R2 binds upstream of the insertion site. (**Materials and Methods** and **Table S3**). In our test case, we used an empty plasmid and successfully added three ORFs (339 bp, 549 bp, 1155 bp), in one PCR reaction (**Fig. 6A and 6B**).

## Discussion

EMP is a fast, cost efficient method for seamless insertion of DNA fragments (up to 5 kb inserts tested) into any target plasmid (up to 10.2 kb tested). Using this technology, one can replace or add ORFs, tags or other DNA elements in a one-day procedure leaving no scars behind. Importantly, the method is suitable for most insert lengths of practical importance, when protein expression is concerned. In addition EMP cloning also allows the insertion of multiple sequences at once.

As shown in our comparison study EMP cloning is qualitatively and quantitatively superior to RF cloning by generating higher PCR product amounts, higher colony numbers and a higher ratio of positive clones over background. The improvement is most prominent for inserts > 2.5 kb, where EMP cloning is still very reliable while RF cloning is not.

An important feature of EMP cloning is the option to perform both PCRs in one reaction, which is mechanistically not possible with RF cloning. This speeds up the experimental procedure. The downside of one-step EMP is the missing control checkpoint after the 1^st^ EMP PCR, therefore a potential failure cannot be traced easily. Although one-step EMP is an attractive option, the standard two-step protocol is probably more reliable since less aberrant amplifications are possible.

The strongest advantage of EMP over contemporary recombination based cloning techniques like SLIC is the possibility to monitor the success of vector-insert fusion prior to transformation: a potential failure can be corrected at an earlier stage, saving time. Furthermore the intensity of the product band provides an estimate of the resulting clone efficiency and thus of the number of clones necessary for successful screening.

The high flexibility in choice of insert and vector, seamless insertion and the high efficiency of EMP cloning make this method an ideal tool for any application that requires the generation of plasmid libraries, for example expression libraries for structural biology. Last but not least, EMP cloning is automatable and should be easily applicable to high-throughput efforts.

## Supporting Information

Figure S1 **Agarose gels of 2^nd^ EMP and 2^nd^ RF PCRs of the 10 test cases.** Products of 2^nd^ EMP and 2^nd^ RF PCRs are shown on agarose gels. Product lengths are indicated in parenthesis. The 2^nd^ PCRs of case 3 are shown twice since RF only worked on the second attempt.
(TIF)

Figure S2 **Control digests of the 10 test cases.** Agarose gels of control digests of the 10 test cases are shown. If 10 or more colonies were obtained in an experiment, 10 plasmids were isolated and digested with appropriate restriction enzymes. If less then 10 colonies were obtained, all colonies were analyzed. Circles around colony numbers indicate clones with the expected band patterns. Sizes of the expected products from restriction analysis are indicated to the left of the gels.
(TIF)

Figure S3 **Colony PCR of one-step EMP reaction.** Agarose gel of colony PCRs of 7 clones obtained by one-step EMP. 4 out of 7 clones show the right PCR product at 948 bp.
(TIF)

Table S1 **Exponential megapriming PCR protocol.**
(TIF)

Table S2 **One-step EMP PCR protocol.**
(TIF)

Table S3 **Multi-insert EMP PCR protocol.**
(TIF)

Table S4 **Restriction-free cloning PCR protocol.**
(TIF)
