# Correction: Exponential Megapriming PCR (EMP) Cloning—Seamless DNA Insertion into Any Target Plasmid without Sequence Constraints

**DOI:** 10.1371/annotation/7465cec8-d898-4d11-b32d-fcd1a8c24f25

**Published:** 2013-09-20

**Authors:** Alexander Ulrich, Kasper R. Andersen, Thomas U. Schwartz

An error was introduced in the preparation of this article for publication. The error is in the penultimate sentence of the "One-step EMP Cloning" section of the Materials and Methods. 

The correct sentence is: A typical reaction contains 0.02 μM primer R1, 0,5 μM primer F1 and 0.5 μM primer R2 in addition to 1x HF Phusion buffer, 200 μM of each dNTP, 25 ng template DNA for the insert, 25 ng template DNA for the target plasmid, and 0.02 U/μL Phusion DNA Polymerase (NEB) in a 50 μL reaction. 

