# Supplementary figures and images for: Exponential Megapriming PCR (EMP) Cloning—Seamless DNA Insertion into Any Target Plasmid without Sequence Constraints
Source: PLoS One. 2012 Dec 31;7(12):e53360. doi: 10.1371/journal.pone.0053360 (PMC3534072; doi:10.1371/journal.pone.0053360)

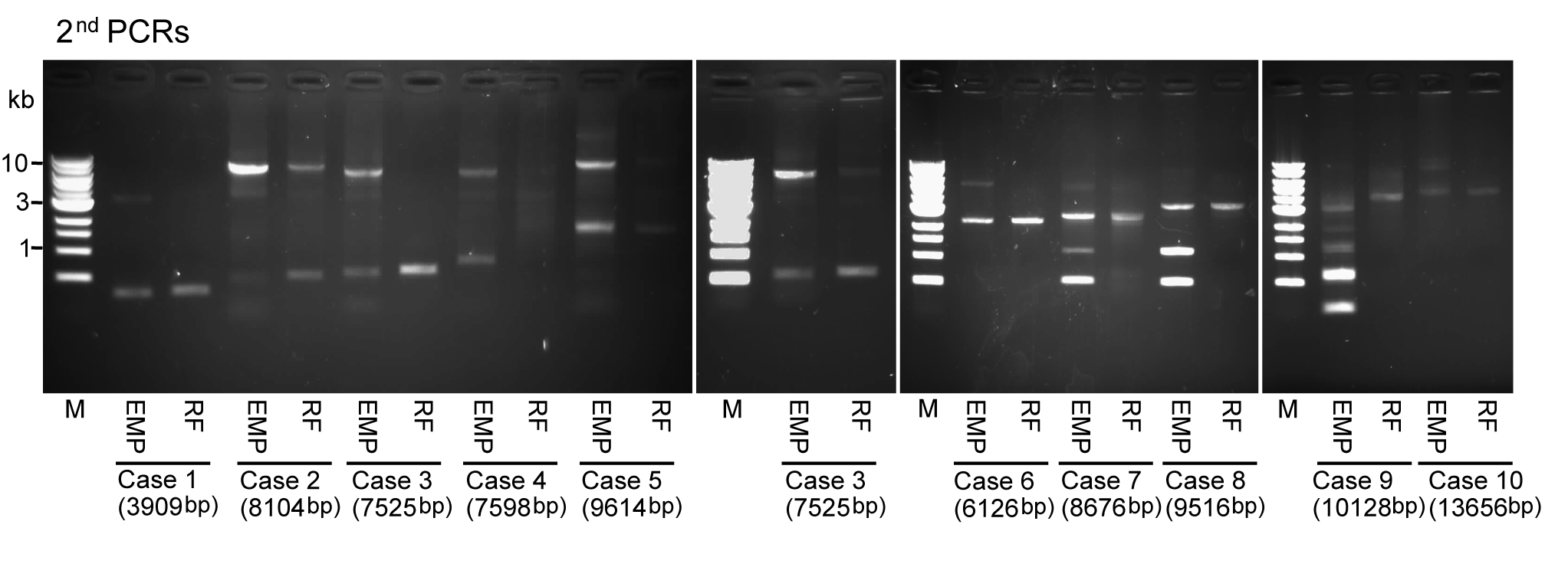

Supplement: Figure S1 — Agarose gels of 2nd EMP and 2nd RF PCRs of the 10 test cases. Products of 2nd EMP and 2nd RF PCRs are shown on agarose gels. Product lengths are indicated in parenthesis. The 2nd PCRs of case 3 are shown twice since RF only worked on the second attempt. (TIF) [file pone.0053360.s001.tif]

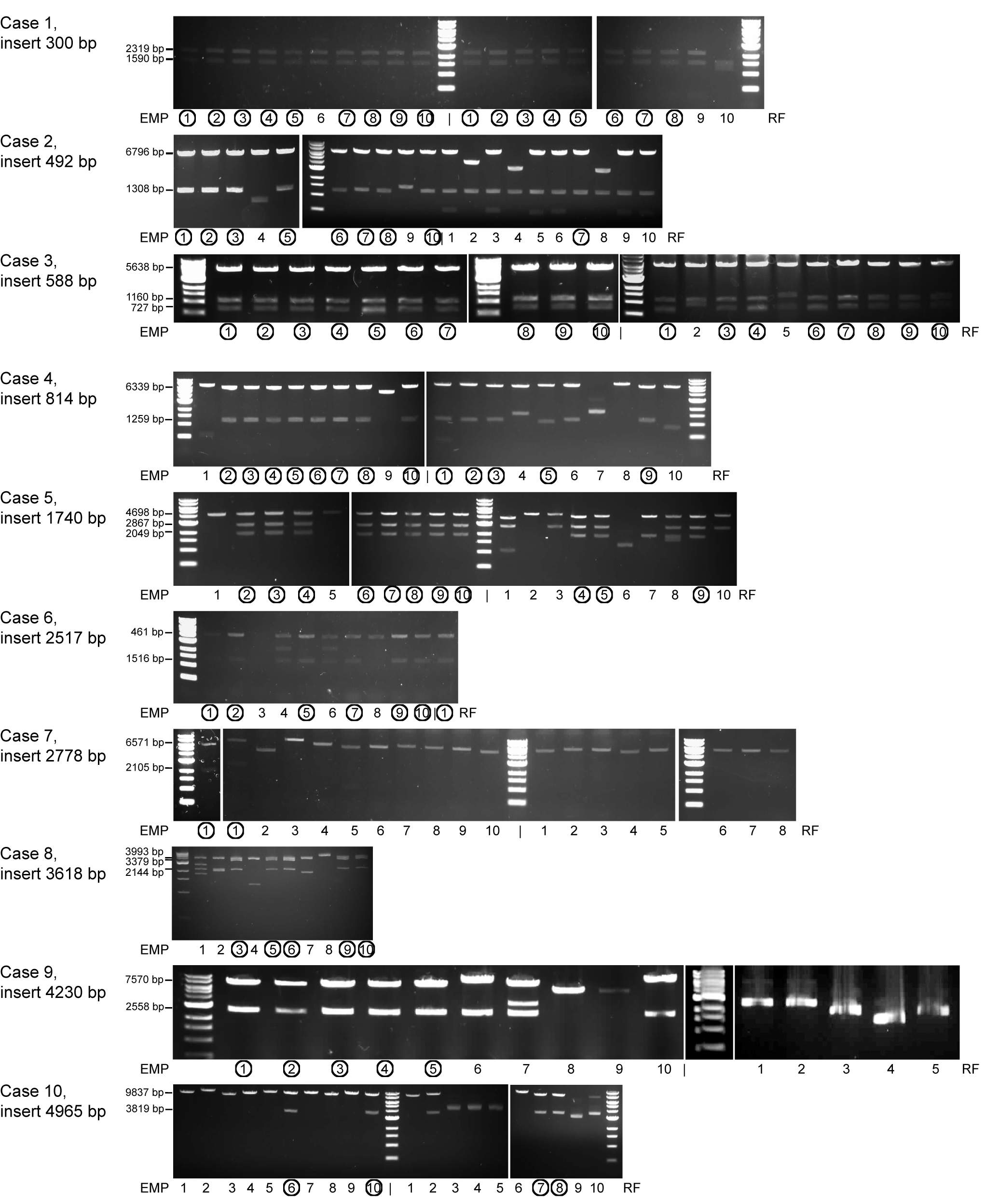

Supplement: Figure S2 — Control digests of the 10 test cases. Agarose gels of control digests of the 10 test cases are shown. If 10 or more colonies were obtained in an experiment, 10 plasmids were isolated and digested with appropriate restriction enzymes. If less then 10 colonies were obtained, all colonies were analyzed. Circles around colony numbers indicate clones with the expected band patterns. Sizes of the expected products from restriction analysis are indicated to the left of the gels. (TIF) [file pone.0053360.s002.tif]

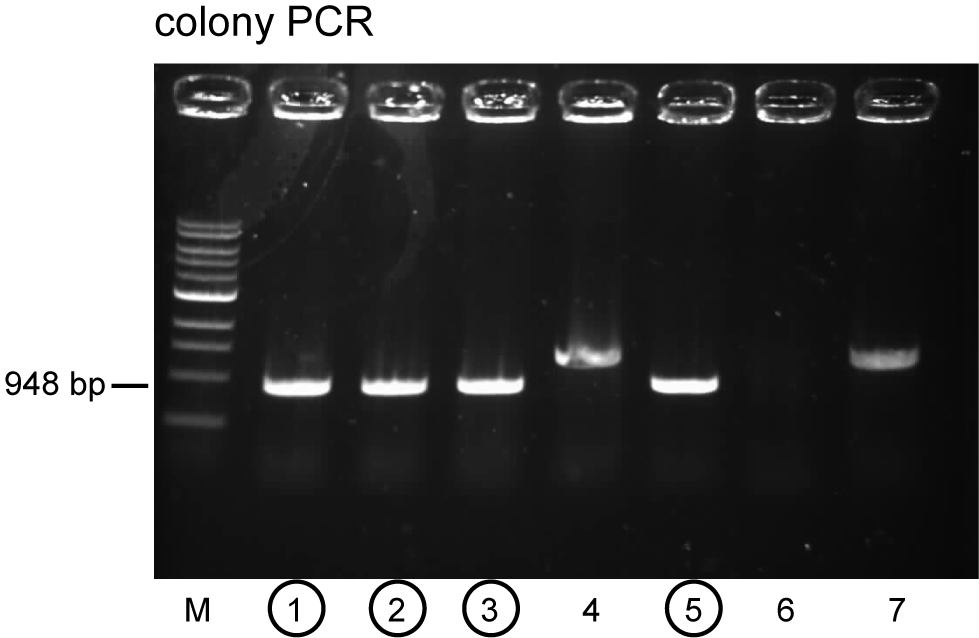

Supplement: Figure S3 — Colony PCR of one-step EMP reaction. Agarose gel of colony PCRs of 7 clones obtained by one-step EMP. 4 out of 7 clones show the right PCR product at 948 bp. (TIF) [file pone.0053360.s003.tif]

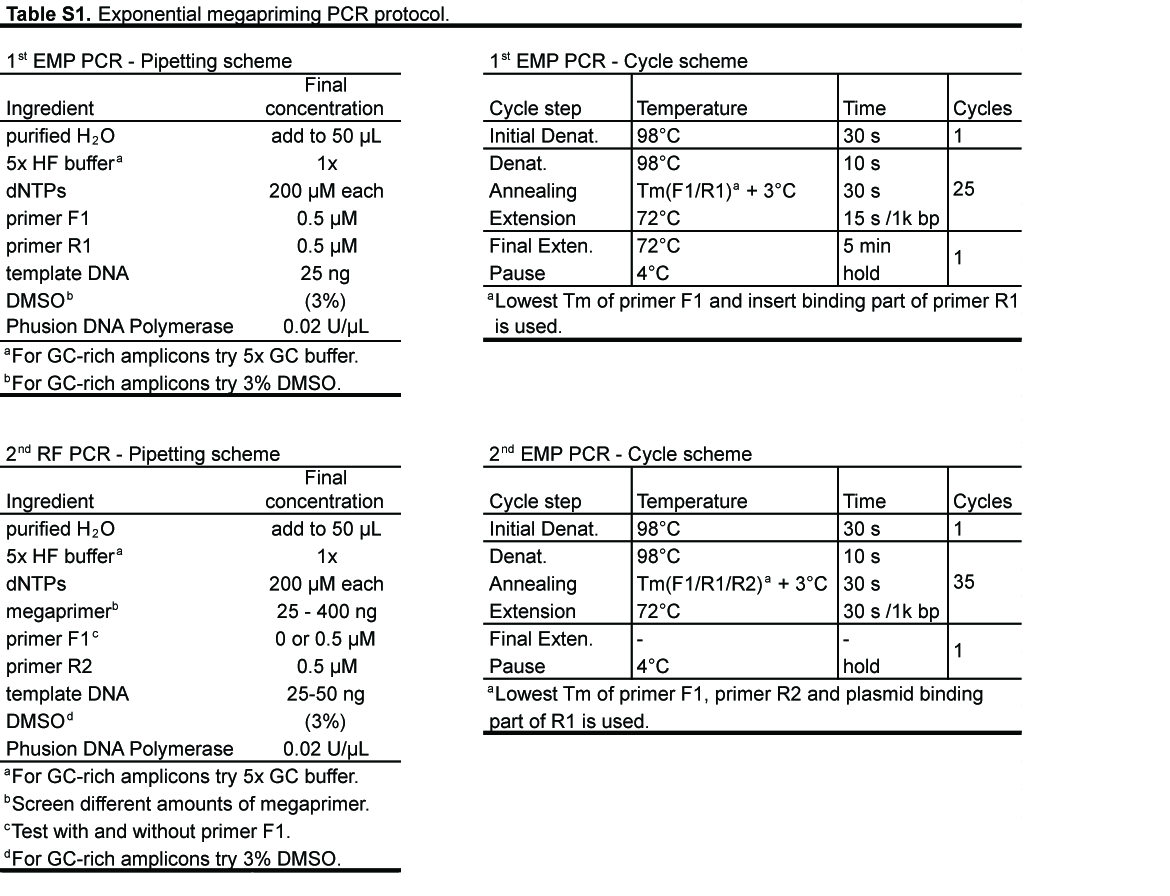

Supplement: Table S1 — Exponential megapriming PCR protocol. (TIF) [file pone.0053360.s004.tif]

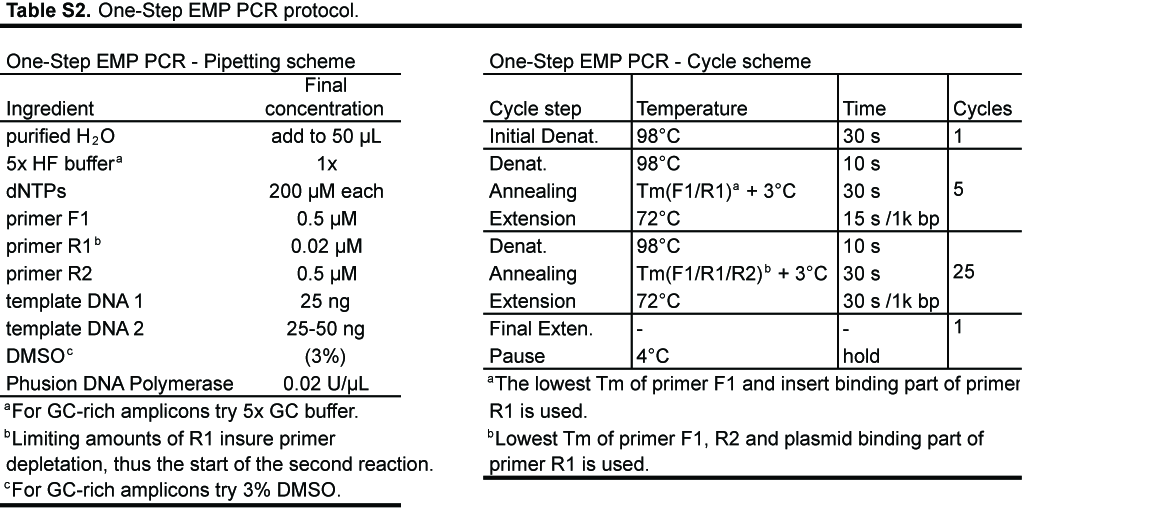

Supplement: Table S2 — One-step EMP PCR protocol. (TIF) [file pone.0053360.s005.tif]

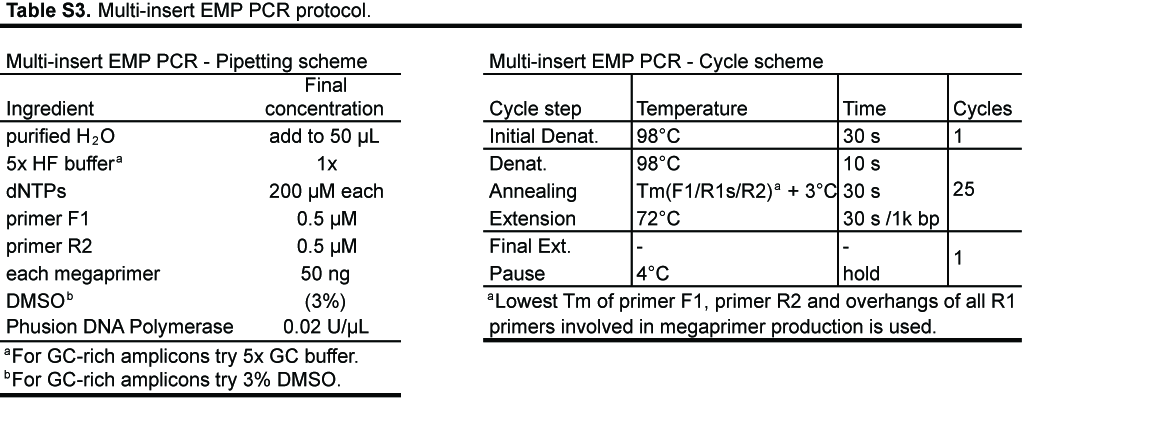

Supplement: Table S3 — Multi-insert EMP PCR protocol. (TIF) [file pone.0053360.s006.tif]

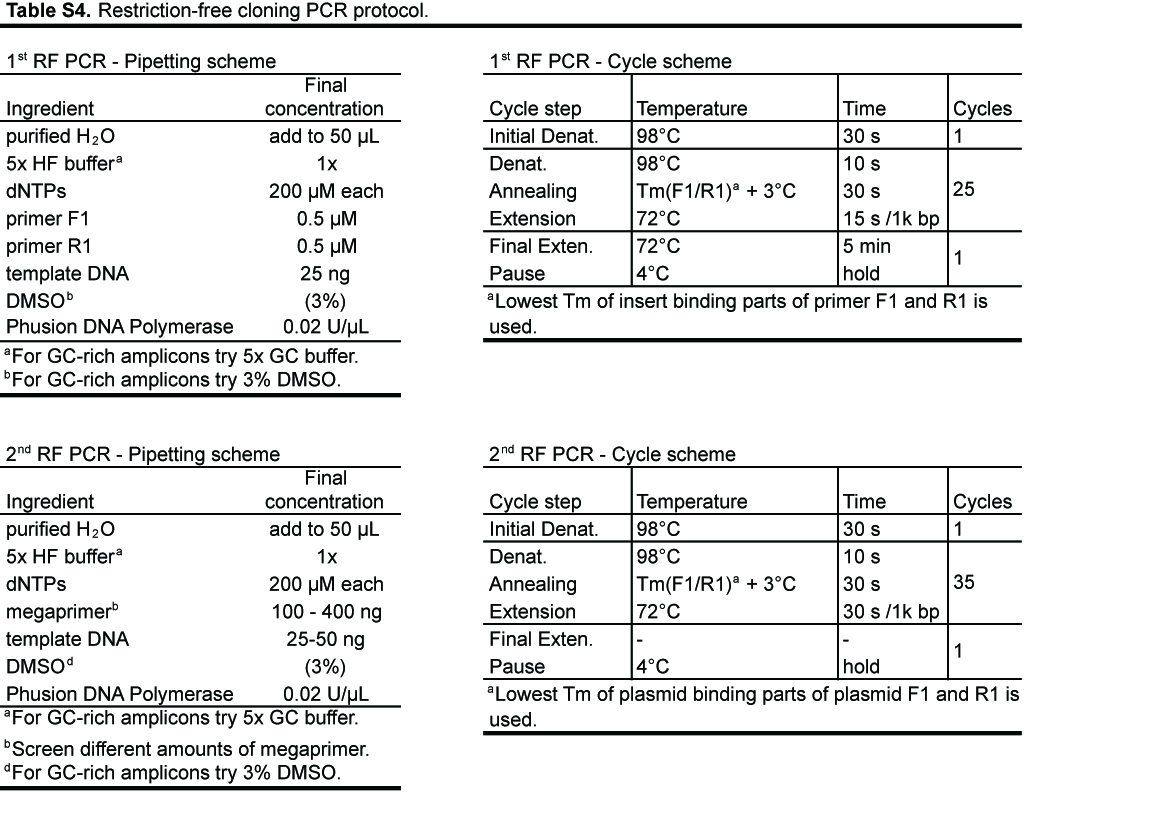

Supplement: Table S4 — Restriction-free cloning PCR protocol. (TIF) [file pone.0053360.s007.tif]
